# Supervised Learning Methods for Modeling Concrete Compressive Strength Prediction at High Temperature

**DOI:** 10.3390/ma14081983

**Published:** 2021-04-15

**Authors:** Mahmood Ahmad, Ji-Lei Hu, Feezan Ahmad, Xiao-Wei Tang, Maaz Amjad, Muhammad Junaid Iqbal, Muhammad Asim, Asim Farooq

**Affiliations:** 1 Department of Civil Engineering, University of Engineering and Technology Peshawar (Bannu Campus), Bannu 28100, Pakistan; ahmadm@uetpeshawar.edu.pk (M.A.); engrjunaidiqbal@uetpeshawar.edu.pk (M.J.I.); muhammadasim@uetpeshawar.edu.pk (M.A.); 2 College of Civil Engineering and Architecture, China Three Gogres University, Yichang 443002, China; 3 State Key Laboratory of Coastal and Offshore Engineering, Dalian University of Technology, Dalian 116024, China; ahmadf@mail.dlut.edu.cn (F.A.); tangxw@dlut.edu.cn (X.-W.T.); 4 Department of Civil Engineering, University of Engineering and Technology, Peshawar 25120, Pakistan; maazamjad.civ@uetpeshawar.edu.pk; 5 Department of Transportation Engineering, Pak-Austria Fachhochschule: Institute of Applied Sciences and Technology, Haripur 22620, Pakistan; asimfarooq@cecos.edu.pk

**Keywords:** compressive strength, concrete, prediction, data mining, high temperature, sensitivity analysis

## Abstract

Supervised learning algorithms are a recent trend for the prediction of mechanical properties of concrete. This paper presents AdaBoost, random forest (RF), and decision tree (DT) models for predicting the compressive strength of concrete at high temperature, based on the experimental data of 207 tests. The cement content, water, fine and coarse aggregates, silica fume, nano silica, fly ash, super plasticizer, and temperature were used as inputs for the models’ development. The performance of the AdaBoost, RF, and DT models are assessed using statistical indices, including the coefficient of determination (R^2^), root mean squared error-observations standard deviation ratio (RSR), mean absolute percentage error, and relative root mean square error. The applications of the above-mentioned approach for predicting the compressive strength of concrete at high temperature are compared with each other, and also to the artificial neural network and adaptive neuro-fuzzy inference system models described in the literature, to demonstrate the suitability of using the supervised learning methods for modeling to predict the compressive strength at high temperature. The results indicated a strong correlation between experimental and predicted values, with R^2^ above 0.9 and RSR lower than 0.5 during the learning and testing phases for the AdaBoost model. Moreover, the cement content in the mix was revealed as the most sensitive parameter by sensitivity analysis.

## 1. Introduction

Concrete is one of the most versatile materials used in the construction of buildings, subway systems, and many other civil engineering structures. With the rapid development of urbanization, the demand for structural concrete is increasing. As a core aspect of these structures, concrete may encounter aberrant results such as abrasion, freezing, and chemical erosion during the whole life of the structure. One of the aberrant results is high temperature and fire. Some examples of concrete structure that are vulnerable to high temperature include industrial structures, such as chimneys working at high temperature, as well as factories dealing with chemicals with high fire risk [1]. The fire causes the concrete temperature in the concrete structure to be extremely high. If the concrete surface reaches above 100 °C, it can be observed that heat transfer can increase the internal temperature of concrete to 300–700 °C [2].

Concrete is a non-combustible material, but when subjected to high temperatures, its chemical, physical, and mechanical properties change with the impact of high temperatures [3]. Chemical and physical reactions in hot concrete, such as dehydration, decomposition [4,5], and rapid increase in vapor pressure and thermal stress, result in concrete spillage, cracking, and perforation, resulting in a deterioration of the mechanical properties of concrete [6]. Tanyildizi [7] showed that as the temperature of concrete increased, the width and length of cracks increased as well. Despite the fact that disasters such as fire or explosion do not cause direct damage, such events may, in the long or short term, damage the structure’s stiffness or structural strength [8].

There is a direct relationship between the temperature increase and the decrease in the compressive strength of concrete, according to National Institute of Standards and Technology (NIST) Technical Note 1681 [9]. In this guideline, concrete with a compressive strength less than 83 MPa is referred to as normal-strength concrete (NSE), and concrete with a compressive strength greater than 83 MPa is referred to as high-strength concrete (HSC). The relationship between concrete compressive strength and temperature was studied by Malhotra [10], who found a relationship between temperature increasing and concrete strength decreasing. Numerous variables can influence the actual behavior of concrete at high temperatures, such as the properties of the constituent concrete materials, the rate of increase in temperature, and the maximum temperature [11].

Deterioration of concrete exposed to high temperatures is attributed to three factors: physicochemical changes in the cement paste, aggregates, and the thermal incompatibility between them. Concrete deterioration is influenced by fire-related factors such as temperature and heating rate, as well as structural element conditions such as applied load and humidity [12]. As a result, it is critical to discuss the effects of high temperatures on concrete, with a focus on aggregate microstructural changes, hydrated cement paste, and the transition zone. The transformations that occur before the temperature reaches 1200 °C—at which concrete begins to melt—will be investigated [12]. It is worth noting that real fire can reach temperatures of over 900 °C; however, it is limited to the surface layers of structural elements, with the internal temperature remaining relatively low [13].

Several studies in the literature have investigated the numerical analysis of concrete exposed to high temperatures, such as Ožbolt et al. [14], who investigated 3D thermo-mechanical numerical analysis of concrete beams that had been exposed to high temperatures. For high temperature concrete failure analysis, a coupled thermo-mechanical interface model was used by Caggiano and Etse [15]. In the interface model, the coupled thermal-mechanical effect was taken into account by formulating a temperature-dependent maximum strength criterion and a fracture-energy-based softening or post-cracking law. A model of the elasto-thermo-plastic interface was proposed in this way to simulate the behavior of concrete cracking and failure. The surface of the cross-section exposed to high temperatures heats up rapidly, but the inner sections of the cross-section have slightly lower temperatures. Restrained stresses cause the concrete to crack as a result of such temperature gradients [14]. It is important to note that the weakening of the strength criterion is strictly related to the cracking of concrete due to temperature effects. The disadvantage of numerical modeling is the complexity of the model preparation, the numerical modeling calculations, and the evaluation of the results.

Machine learning (ML), which includes supervised learning methods, are an increasing trend in various fields for the prediction of different properties. Similarly, the civil engineering construction industry has also adopted such techniques for the prediction of mechanical properties of concrete to overcome cumbersome experimental procedures. The artificial neural network (ANN) method was employed by Trtnik et al. [16] to measure the compressive strength of concrete. It has been shown that the experimental values are correctly expressed by ANN; hence, it proves to be an exceptional prediction method. Keshavarz et al. [17] predicted the compressive strength of concrete with ANN and adaptive neural-fuzzy inference system (ANFIS) models. The authors show that ANFIS offers a more generalized and better correlation than the ANN model. By performing an experimental and literature-based analysis, Javed et al. [18] predicted the compressive strength of sugar cane bagasse ash concrete. Hadzima-Nyarko et al. [19,20] investigated ANN, k-nearest neighbor, regression trees, and random forests models for predicting the compressive strength of concrete. Zhang et al. [21] developed a model that combines beetle antennae search (BAS) and multi-output least square support vector regression (MOLSSVR) to predict concrete compressive strength and pervious permeability coefficient. Their proposed model outperforms support vector regression (SVR), MOLSSVR, logistic regression, and modified ANN with firefly algorithm, according to the findings of this study. To estimate the compressive strength of concrete with ground granulated blast-furnace slag, Kandiri et al. [22] developed a hybrid ANN with multi-objective salp swarm algorithm. Golafshani et al. [23] showed that an ANN-based model was more effective than modified ANFIS by combining ANFIS and ANN with the grey wolf optimizer. Ali Khan et al. [24] used gene expression programming (GEP) for prediction of the compressive strength of geopolymer concrete (GPC), and found that the GEP model possesses a higher predictive capability and is appropriate to practice in the preliminary design of fly-ash-based GPC. The results showed that the aforementioned ML models re able to obtain the experimental observations with an acceptable performance. However, this field continues to be further explored.

This paper focuses on the use of computational intelligence techniques—especially AdaBoost, random forest (RF), and decision tree (DT) algorithms—to analyze the prediction of concrete’s compressive strength at high temperature, emphasizing accuracy and efficiency, and each technique’s potential to deal with experimental data. This study also aims to contribute to the knowledge of the application of computational models in the prediction of compressive strength of concrete at high temperature, using machine learning and comparing the obtained results with other studies in the available literature. The primary significance of this study is that the data division in the training and testing datasets has been made with due regard to statistical aspects such as maximum, minimum, mean, and standard deviation. The splitting of the datasets is made to determine the predictive capability and generalization performance of established models, and it later helps to better evaluate them. Finally, a sensitivity analysis is also carried out on input parameters.

The rest of this article is structured as follows: The next section introduces the data catalog and the selection of input variables. Section 3 presents the preliminaries of the algorithms used in the proposed approach, and discusses the model evaluation metrics. Development of AdaBoost, RF, and DT of proposed models are described in Section 4. Section 5 describes the results and discussion. Finally, Section 6 draws conclusions and outlines promising directions for future work.

## 2. Data Catalog and Input Variables Selection

The data used in the study comprise a total of 207 experimental results on the residual compressive strength from the synthesis of previously published “source catalogs.” The source catalogs are those of Ergün et al. [3], Cülfik and Özturan [25], Behnood and Ziari [26], Bastami et al. [27], Chen et al. [28], Xiong et al. [29], Mousa [30], Fu et al. [31], and Husem [32]. The data catalog is presented in Table 1 (the entire database can be found in Appendix A, Table A1).

It should be noted that the samples that were chosen from the mentioned references were taken at the age of 28 days. In this study, 165 (80%) and 42 (20%) samples were selected based on statistical consistency—such as minimum (Min.), maximum (Max.), and mean—for the training and testing, respectively, of the proposed models. The statistical consistency of the datasets for training and testing optimizes the performance of the models and eventually helps to analyze them better.

A significant step in predictive modeling in data mining is the selection of the input variables that represent the system to be modelled. The input variables of a data-driven model should contain all relevant information about the target output. On the other hand, they rely to a large extent on the information available in the form of input-output data pairs. The proportions of the mix, the temperature, and the compressive strength associated with the temperature are the data available from the literature related to concrete’s compressive strength when exposed to high temperature. Consequently, the temperature proportions of the mix (the quantity of different materials, such as water, cement, fine and coarse aggregates, and admixtures) may be the correct choice of the input variables to predict the compressive strength of concrete for 28 days at any temperature. In this study, the input variables are cement (C), water (W), fine aggregates (FA), coarse aggregates (CA), silica fume (SF), nano silica (NS), fly ash (F), super plasticizer (SP), and temperature (T); and the output variable is compressive strength of concrete at the temperature T (*f**’_c,T_***). The descriptive statistics of each input and output are listed in Table 2.

## 3. Methodology

In this section, each of the algorithms used in the proposed methodology is briefly described.

### 3.1. AdaBoost

AdaBoost is a commonly used boosting algorithm that constructs an ensemble by performing multiple iterations each time with different instance weights, and adapts to the errors returned by classifiers from previous iterations [33,34] adaptively. In each iteration, changing the weight of training instances forces the learning algorithms to put more emphasis on instances previously incorrectly classified, and less emphasis on instances previously correctly classified. In other words, for misclassified instances, weights are increased, while for correctly classified instances, weights are reduced. This will make sure that misclassification errors count more heavily in the next iterations for those misclassified instances. AdaBoost utilizes the predictions of several weak classifiers, and a final prediction is given by a combined vote on techniques.

The principal concept of the AdaBoost learning algorithm is to create a strong classifier that has high performance detection by joining weak classifiers. The AdaBoost algorithm learns, and has two functions with repetitive calculations: selecting the function and learning the classifier. By reiterating the calculation, the simple classifying ability is strengthened, because weak classifiers that are an index of performance are added to the powerful iteration classifier [35].

### 3.2. Random Forest

**Figure 1:**
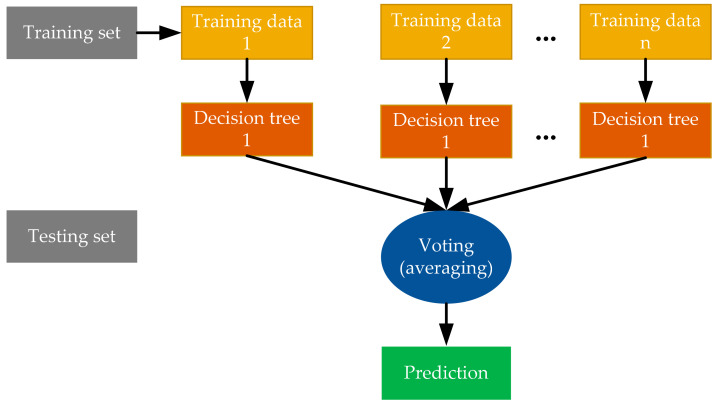
Schematic illustration of RF structure.

Random forest (RF) is a supervised learning algorithm which is used for both classification and regression. It is, however, primarily used for problems with classification. Breimanan presented the theoretical development of RF [36]. On data samples, the RF algorithm generates decision trees and then gets the prediction from each of them and eventually chooses the best solution by voting. It is an ensemble approach that is better than a single decision tree because by averaging the result, it decreases the over-fitting. An RF algorithm’s working procedure consists of the following steps:

Step 1—Start by selecting random samples from a given dataset.
Step 2—Next, for each sample, this algorithm creates a decision tree. Then, from any decision tree, it gets the prediction result.
Step 3—For any predicted outcome, voting is carried out in this step.
Step 4—Eventually, the outcome of the most voted prediction is selected as the final result of the prediction. Figure 1 presents RF working architecture.

### 3.3. Decision Tree

The decision tree (DT) constructs classification or regression models in the form of a tree diagram. It divides a dataset into smaller and smaller subsets while simultaneously building an associated decision tree [37]. To predict a class label for a record in DT, we start at the root of the tree. The values of the root attribute are compared to the attributes of the record. We jump to the next node after following the branch of this value based on the relation. Decision trees classify instances by sorting the tree from the root to a specific leaf or terminal node and supplying the instance classification to the leaf node. Every tree node serves as a test case for a specific attribute, and the possible responses to the test case correspond with each edge descending from the node. This is a recursive method that is replicated for all sub-trees that are rooted in the new node. A minimum number of leaf instances, splitting into smaller subsets, a maximum number of depths, and stopping nodes from splitting before the required majority threshold has been reached are all included in the DT parameters.

## 4. Construction of Prediction Models

**Figure 2:**
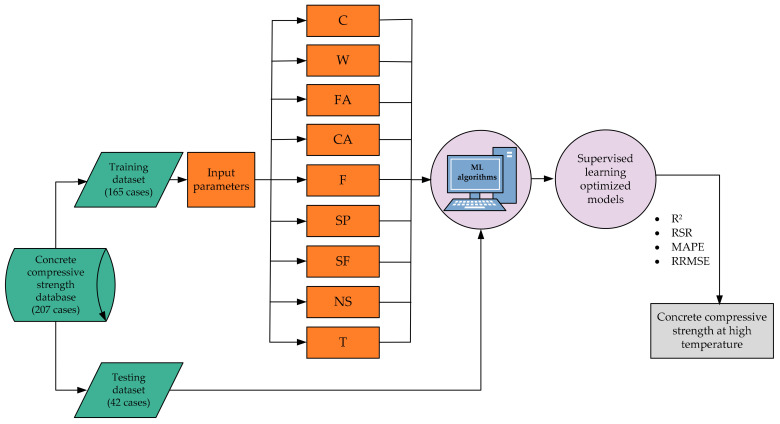
Framework of the proposed study.

The proposed models for prediction of the compressive strength of concrete at high temperature (*f’_c,T_*) were developed using Orange software. The model structure was based on an input matrix (*x*) defined by *x* = [C, W, FA, CA, F, SP, SF, NS, and T], which provided the predictor variables, while the target variable (*y*) was *f’_c,T_*. The most important task is to find the acceptable size of the training data and testing dataset in every modeling phase. Therefore, 80% of the total dataset was selected and used to create the models in this analysis, and the developed models were evaluated on the remaining dataset. In other words, to build and evaluate the models, 165 and 42 datasets were used, respectively. Based on the trial and error process, all models (AdaBoost, RF, and DT) were tuned in order to optimize the *f’_c,T_* prediction. The construction of the prediction models is shown in Figure 2.

### 4.1. Hyperparameter Optimization

Hyperparameters that need to be tuned are found in most of the ML algorithms. In order to obtain the best prediction accuracy, the optimization process aims to determine the best parameters for AdaBoost, RF, and DT. In this research, as shown in Table 3, some critical hyperparameters in AdaBoost, RF, and DT algorithms are tuned. Table 3 also clarifies the specific meanings of these hyperparameters. First, the values for the tuning parameters of the models were selected, and then subsequently varied in the trials until the best fitness measures provided in Table 3 were achieved.

### 4.2. Model Evaluation Indexes

The coefficient of determination (R^2^), root mean square error (RMSE)-observations standard deviation ratio (RSR), mean absolute percentage error (MAPE), and relative root mean square error (RRMSE), as more common criteria in the literature, are used in this study to evaluate the results of the proposed models.

(1) $$R^{2}=1-\frac{\sum_{i=1}^{n}{\left(y_{i}-{\hat{y}}_{i}\right)}^{2}}{\sum_{i=1}^{n}{\left(y_{i}-\hat{y}\right)}^{2}}$$

(2) $$RSR=\frac{\sqrt{\sum_{i=1}^{n}{\left(y_{i}-{\hat{y}}_{i}\right)}^{2}}}{\sqrt{\sum_{i=1}^{n}{\left(y_{i}-\bar{y}\right)}^{2}}}$$

(3) $$MAPE=\frac{1}{n}\sum_{i=1}^{n}\left|\left.\frac{y_{i}-{\hat{y}}_{i}}{y_{i}}\right|\right.\times 100\%$$

(4) $$RRMSE=\sqrt{\frac{\sum_{i=1}^{n}{\left(y_{i}-{\hat{y}}_{i}\right)}^{2}}{n{\left(\bar{y}\right)}^{2}}}\times 100\%$$

In the equations, *n* is the number of data; *y_i_* and ${\hat{y}}_{i}$ are the actual and predicted output of *i*th sample of the data, respectively; $\bar{y}$ is the averaged actual output of the data. The R^2^ coefficient ranges from 0 to 1, and the model a with higher quantity of R^2^ has more efficiency. MAPE and RRMSE criteria measure the percentage error of the model in two different forms, which range from 0 to 100. The model is deemed effective when the value of R^2^ is greater than 0.8 and is close to 1 [38]. The RMSE-observations standard deviation ratio (RSR) is calculated as the ratio of the RMSE and standard deviation of measured data. The RSR varies from an optimal value of 0 to a significant positive value. A lower RSR presents a lower RMSE, indicating the higher predictive efficiency of the model. RSR classification ranges are described as very good, good, acceptable, and unacceptable with ranges of 0.00 ≤ RSR ≤ 0.50, 0.50 ≤ RSR ≤ 0.60, 0.60 ≤ RSR ≤ 0.70, and RSR > 0.70, respectively [39]. It is obvious the lower the values of RSR, MAPE, and RRMSE criteria, the better the model.

## 5. Results and Discussion

**Figure 3:**
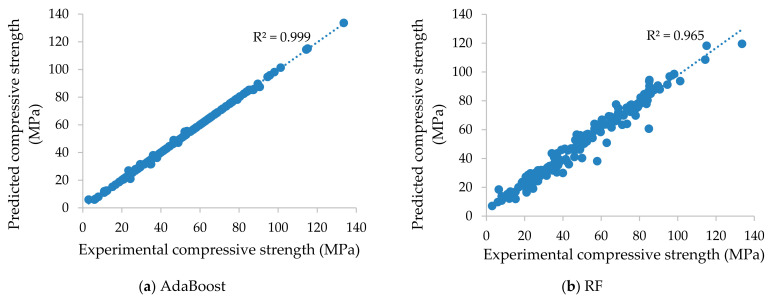

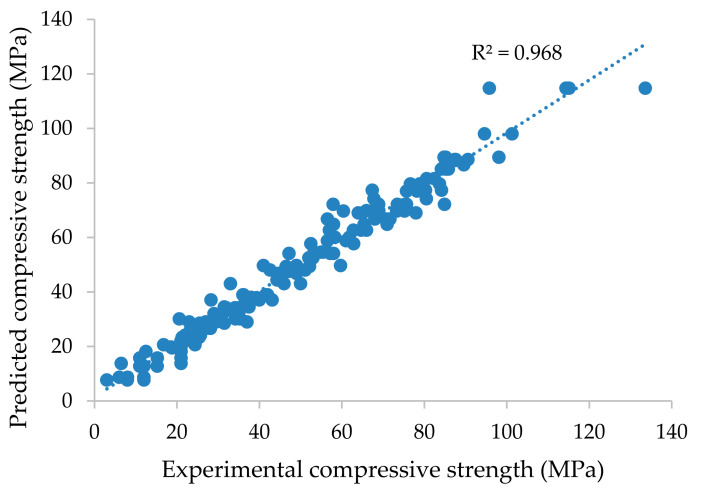
Scatter plots displaying the experimental (actual) compressive values versus the predicted compressive values of concrete at high temperature of the training dataset using the (**a**) AdaBoost, (**b**) RF, and (**c**) DT algorithms.

**Figure 4:**
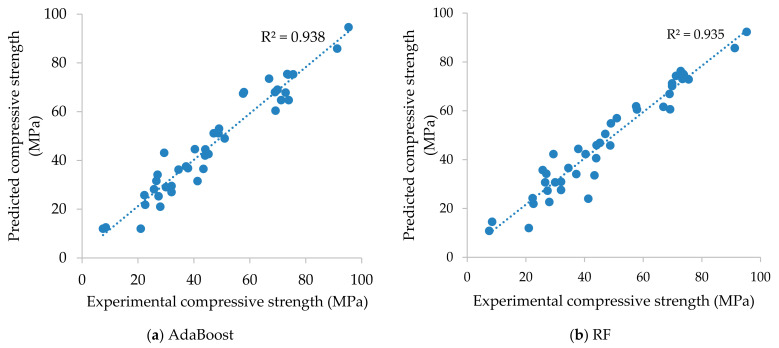

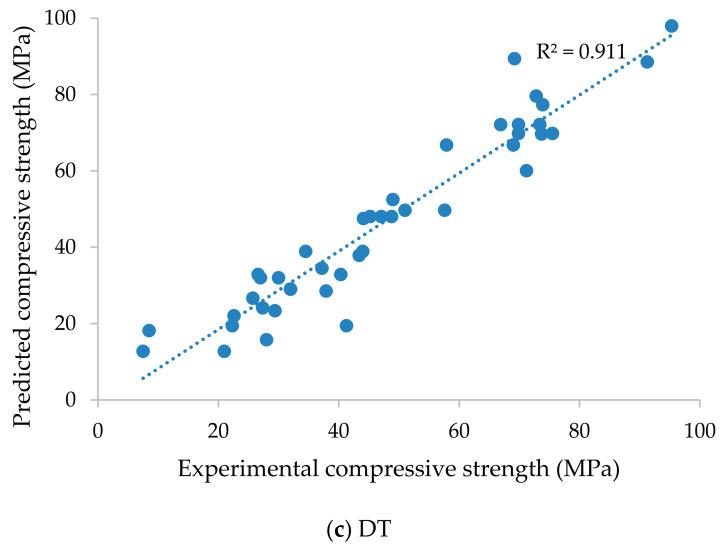
Scatter plot presenting the experimental (actual) compressive values versus the predicted compressive values of concrete at high temperature of the testing dataset using the (**a**) AdaBoost, (**b**) RF, and (**c**) DT algorithms.

In this section, using training and test datasets, the predictive performance of the established AdaBoost, RF, and DT models is assessed. The training dataset is used to assess the model structure and parameters. As a result, the performance of the models on the training dataset can be used to determine which model is well trained. However, the test dataset is used only after the model has been determined to evaluate the quality of the model. According to the predicted values, the values of the different statistical measures of the models for both the training and test phases are shown in Table 4 and Table 5. Figure 3 and Figure 4 display the scatter plot of the experimental (actual) and the predicted compressive strength of concrete at high temperature involving three supervised learning techniques for the phases of training and testing. From these findings, it is clear that, in terms of statistical performance measures, all models performed effectively in predicting the compressive strength of concrete at high temperatures. In the training dataset, the R^2^ was determined to be higher in the case of AdaBoost (R^2^ = 0.999) as compared with the other two models, RF (R^2^ = 0.965) and DT (R^2^ = 0.968). Similarly, in the testing phase, the R^2^ was determined to be higher in the case of AdaBoost (R^2^ = 0.938) as compared with the other two models, RF (R^2^ = 0.935) and DT (R^2^ = 0.911)

Furthermore, in terms of the statistical measures in training, the lowest value was found for AdaBoost (RSR = 0.032, MAPE = 1.357%, RRMSE = 1.666%) compared to RF (RSR = 0.190, MAPE = 11.306%, RRMSE = 9.869%) and DT (RSR = 0.178, MAPE = 9.747%, RRMSE = 9.265%). Similarly, regarding the prediction results in the testing, the lowest value was found for AdaBoost (RSR = 0.248, MAPE = 12.523%, RRMSE = 11.622%) compared to RF (RSR = 0.256, MAPE = 13.076%, RRMSE = 11.661%) and DT (RSR = 0.324, MAPE = 16.100%, RRMSE = 14.753%). This superiority may be owing to the fact that the AdaBoost model excellently captures the nonlinear relationships between concrete mix proportions and temperature with compressive strength. It can therefore be concluded that, based on statistical analysis checks, the AdaBoost model had the best results.

Additionally, the R^2^, MAPE, and RRMSE of the predicted values using the ANFIS method [40] were 0.94, 14%, and 13%, respectively, for the training dataset. The R^2^, MAPE, and RRMSE of the predicted values using the ANN method [20] were 0.96, 9%, and 10%, respectively, for the training dataset. Similarly, the R^2^, MAPE, and RRMSE of the predicted values using the ANFIS method [40] were 0.89, 20%, and 15%, respectively, for the testing dataset. The R^2^, MAPE, and RRMSE of the predicted values using the ANN method [40] were 0.92, 12%, and 12%, respectively, for the testing dataset. The performance has been improved by the AdaBoost model compared with the ANFIS and ANN models in terms of R^2^, MAPE, and RRMSE values. In particular, the AdaBoost model yielded the best result in the section of training and testing datasets. 

Finally, it can be seen, the performance accuracy of the AdaBoost model is higher than the RF and DT models. In general, this study may assist engineers in selecting appropriate supervised learning models and parameters for the production of high-temperature concrete.

**Figure 5:**
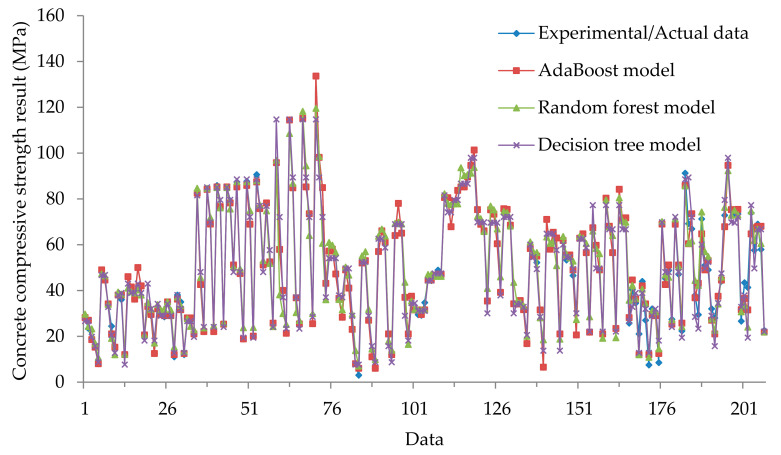
Compressive strength results of AdaBoost, RF, and DT models in training and testing phases.

The values obtained from the three models and the experimental values are presented in Figure 5. It can be inferred from this figure that using the AdaBoost model might be sufficient and have reasonable precision with nine input variables for the estimation of the compressive strength of concrete at high temperature. Based on the findings, using a set of nine input variables could be justifiable and useful for practical and engineering applications.

**Figure 6:**
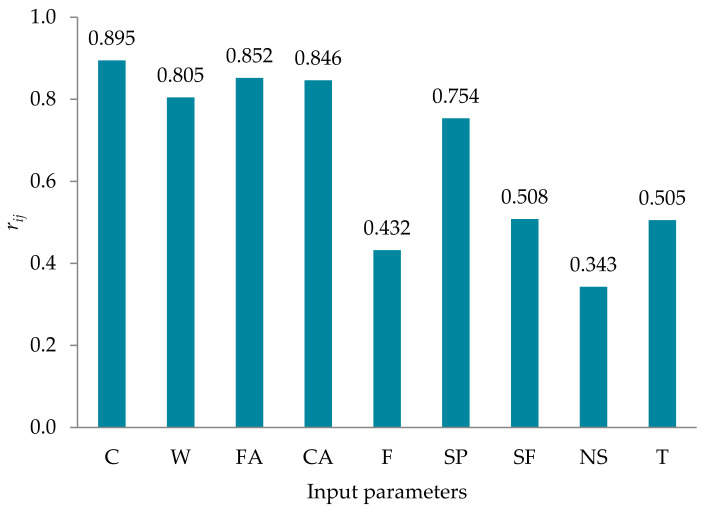
Sensitivity analysis results.

Furthermore, a sensitivity analysis was also conducted using Yang and Zang’s [41] method to evaluate the influence of input parameters on *f’_c,T_* based on the AdaBoost algorithm. This approach has been used in several studies [42,43,44], and is formulated as

(5) $$r_{ij}=\frac{\sum_{m=1}^{n}\left(y_{im}\times y_{om}\right)}{\sqrt{\sum_{m=1}^{n}y_{im}{}^{2}\sum_{m=1}^{n}y_{om}{}^{2}}}$$

where *n* is the number of data values (this study used 165 data values), and *y_im_* and *y_om_* are the input and output parameters. The *r_ij_* value ranged from zero to one for each input parameter, and the highest *r_ij_* values suggested the most efficient output parameter (which was *f’_c,T_* in this study). The *r_ij_* values for all input parameters are presented in Figure 6. The cement (C) content (*r_ij_* = 0.895) in the mix was revealed as the most sensitive parameter, followed by FA (*r_ij_* = 0.852), CA (*r_ij_* = 0.846), W (*r_ij_* = 0.805), SP (*r_ij_* = 0.754), SF (*r_ij_* = 0.508), T (*r_ij_* = 0.505), F (*r_ij_* = 0.432), and NS (*r_ij_* = 0.343), by sensitivity analysis. 

Similar to other artificial intelligence techniques, supervised learning models have a limited domain of applicability and are mostly case dependent. Therefore, their generalization is limited, and they are only applicable in the range of training datasets. Furthermore, the developed AdaBoost model, in comparison to the other models, is suitable to accurately and efficiently predict the NSC and the HSC compressive strength at high temperature. However, this model can always be updated to yield better results as new data becomes available.

## 6. Conclusions and Future Prospect

Robustness and sensitivity analyses of three supervised learning models (i.e., AdaBoost, RF, and DT) were performed in this study for prediction of the compressive strength of concrete at high temperature. Statistical measure criteria such as R^2^, RSR, MAPE, and RRMSE were used to test the predictive abilities of the aforementioned models. In addition, the developed models were compared with ANIFS and ANN models from the literature in order to evaluate robustness. The testing phase results revealed that the supervised learning models built in this study performed well in predicting concrete compressive strength at high temperature, but the most effective model compared to other supervised learning models was the AdaBoost model (R^2^ = 0.938, RSR = 0.248, MAPE = 12.523%, and RRMSE = 11.622%). Statistical analysis checks reveal that the AdaBoost model shows enhancement in model accuracy by minimizing the error difference between targeted and predicted values. The results of the sensitivity analysis show that five parameters—namely, the cement content, the fine and coarse aggregate, the water, and the super plasticizer—were found to be the most sensitive and important factors for predicting the compressive strength of the concrete at high temperature. It can therefore be inferred that the AdaBoost model is a promising method for predicting concrete compressive strength at high temperature, which can be extended to predict other significant concrete properties, such as elasticity modulus, flexural strength, or tensile strength. Thus, the application of an AdaBoost in the field of predicting the compressive strength at high temperature against destructive testing methods is appropriate, and can be seen as an alternative and suitable approach.

Different artificial intelligence (AI) techniques, such as fuzzy logic, response surface methodology (RSM), support vector machine (SVM) analysis, random forest regression (RFR), recurrent neural network (RNN), may also be applied for a better understanding and predicting of the compressive strength of concrete at high temperature. Furthermore, to improve the performance results of prediction models, more experimental data should be collected in future work.

## Figures and Tables

**Table 1:** Concrete compressive strength catalog.

S. No.	Cement (kg/m^3^)	Water (kg/m^3^)	Sand (kg/m^3^)	Gravel (kg/m^3^)	Fly Ash (kg/m^3^)	Super Plasticizer (kg/m^3^)	Silica Fume (kg/m^3^)	Nano Silica (kg/m^3^)	Temperature (°C)	Compressive Strength (MPa)
1	250	123	417	1681	0	0	0	0	20	28.16
2	250	123	417	1681	0	0	0	0	200	23.4
3	250	123	417	1681	0	0	0	0	400	18.57
…	…	…	…	…	…	…	…	…	…	…
205	465	149	615	1168	0	3.1	30	0	200	69
206	450	149	615	1168	0	3.7	45	0	300	57.9
207	450	149	615	1168	0	3.7	45	0	600	22.6

**Table 2:** Statistics of input and output parameters for the training and testing datasets used in the development of the supervised learning model.

Dataset	Statistical Parameter	Input and Output Parameters									
		C	W	FA	CA	F	SP	SF	NS	T	*f’_c,T_*
Training	Min.	250	123	0	0	0	0	0	0	20	3
	Max.	786	385	1345	1681	150	25.9	150	22.5	1000	133.6
	Mean	437.788	182.307	618.139	1051.794	12.758	8.533	28.636	1.636	344.230	49.795
	Standard deviation	96.690	58.811	314.867	315.342	33.165	7.634	36.912	5.110	289.740	25.985
Testing	Min.	250	123	0	0	0	0	0	0	20	7.5
	Max.	786	385	1345	1681	150	25.9	150	22.5	950	95.3
	Mean	437.286	185.338	578.667	1053.429	12.238	8.769	31.990	2.143	394.952	47.411
	Standard deviation	91.757	64.714	329.096	288.532	33.100	7.538	38.092	5.806	278.991	21.855

**Table 3:** Hyperparameter optimization results.

Algorithm	Hyperparameter	Explanation	Optimal Value
AdaBoost	Number of estimators	Number of trees	50
	Learning rate	It determines to what extent the newly acquired information will override the old information	1
RF	Number of trees	Number of trees in the forest	10
	Do not split subsets smaller than	Smallest subset that can be split	05
DT	Min. number of instances in leaves	Minimum number of samples for split nodes	2
	Do not split subsets smaller than	Forbids the algorithm to split the nodes with less than the given number of instances.	5
	Limit the maximal tree depth	Limit the depth of the classification tree to the number of node levels specified.	100

**Table 4:** Comparison of statistical results obtained from the applied predictive models using training phase with available ANFIS and ANN models.

Models	Statistical Performance Results				Reference
	R^2^	RSR	MAPE (%)	RRMSE (%)	
AdaBoost	0.999	0.032	1.357	1.666	This study
RF	0.965	0.190	11.306	9.869	
DT	0.968	0.178	9.747	9.265	
ANFIS	0.94	−	14	13	[40]
ANN	0.96	−	9	10	

“−” represents that this performance statistic is not included in the reference.

**Table 5:** Comparison of statistical results obtained from the applied predictive models using testing phase with available ANFIS and ANN models.

Models	Statistical Performance Results				Reference
	R^2^	RSR	MAPE (%)	RRMSE (%)	
AdaBoost	0.938	0.248	12.523	11.622	This study
RF	0.935	0.256	13.076	11.661	
DT	0.911	0.324	16.100	14.753	
ANFIS	0.89	−	20	15	[40]
ANN	0.92	−	12	12	

“−” represents that this performance statistic is not included in the reference.

## Data Availability

The data used to support the findings of this study are included within the article.

## Appendix A

**Table A1:** Experimental data catalog.

S. No.	Cement (kg/m^3^)	Water (kg/m^3^)	Sand (kg/m^3^)	Gravel (kg/m^3^)	Fly Ash (kg/m^3^)	Super Plasticizer (kg/m^3^)	Silica Fume (kg/m^3^)	Nano Silica (kg/m^3^)	Temperature (°C)	Compressive Strength (MPa)
1	250	123	417	1681	0	0	0	0	20	28.16
2	250	123	417	1681	0	0	0	0	200	23.4
3	250	123	417	1681	0	0	0	0	400	18.57
4	250	123	417	1681	0	0	0	0	600	15.26
5	250	123	417	1681	0	0	0	0	800	8.01
6	350	172	373	1507	0	0	0	0	20	48.99
7	350	172	373	1507	0	0	0	0	100	44.58
8	350	172	373	1507	0	0	0	0	400	34.12
9	350	172	373	1507	0	0	0	0	600	24.41
10	350	172	373	1507	0	0	0	0	800	15.24
11	500	385	0	820	0	6	0	0	20	38
12	500	385	0	820	0	6	0	0	200	36
13	500	385	0	820	0	6	0	0	800	12
14	450	346.5	0	805	0	6	50	0	20	46
15	450	346.5	0	805	0	6	50	0	200	41.5
16	450	346.5	0	805	0	6	50	0	400	36.2
17	400	308	0	790	0	6	100	0	20	50
18	400	308	0	790	0	6	100	0	400	42
19	400	308	0	790	0	6	100	0	800	21
20	350	269.5	0	775	0	6	150	0	20	33
21	350	269.5	0	775	0	6	150	0	200	29
22	350	269.5	0	775	0	6	150	0	800	12.5
23	400	308	0	1038	0	4.8	0	0	20	32
24	400	308	0	1038	0	4.8	0	0	200	29.5
25	400	308	0	1038	0	4.8	0	0	400	28.5
26	360	277.2	0	1028	0	4.8	40	0	20	35
27	360	277.2	0	1028	0	4.8	40	0	400	29
28	360	277.2	0	1028	0	4.8	40	0	800	11
29	320	246.4	0	1015	0	4.8	80	0	20	38
30	320	246.4	0	1015	0	4.8	80	0	200	35
31	320	246.4	0	1015	0	4.8	80	0	800	12
32	280	215.6	0	1005	0	4.8	120	0	20	28
33	280	215.6	0	1005	0	4.8	120	0	200	27
34	280	215.6	0	1005	0	4.8	120	0	400	21
35	500	135	700	1110	0	14	30	0	20	82.47
36	500	135	700	1110	0	14	30	0	600	42.58
37	500	135	700	1110	0	14	30	0	800	22.03
38	500	135	700	1110	0	15	22.5	7.5	20	84.14
39	500	135	700	1110	0	15	22.5	7.5	400	68.99
40	500	135	700	1110	0	15	22.5	7.5	800	23.39
41	500	135	700	1110	0	16	15	15	20	85.84
42	500	135	700	1110	0	16	15	15	400	76.62
43	500	135	700	1110	0	16	15	15	800	25.28
44	500	135	700	1110	0	18	7.5	22.5	20	85.21
45	500	135	700	1110	0	18	7.5	22.5	400	79.12
46	500	135	700	1110	0	18	7.5	22.5	600	51.11
47	470	135	700	1110	0	16	60	0	20	87.38
48	470	135	700	1110	0	16	60	0	600	47.39
49	470	135	700	1110	0	16	60	0	800	18.82
50	470	135	700	1110	0	18	52.5	7.5	20	87.61
51	470	135	700	1110	0	18	52.5	7.5	400	68.94
52	470	135	700	1110	0	18	52.5	7.5	800	20.06
53	470	135	700	1110	0	20	45	15	20	90.6
54	470	135	700	1110	0	20	45	15	400	75.71
55	470	135	700	1110	0	20	45	15	600	51.12
56	470	135	700	1110	0	22	37.5	22.5	400	78.22
57	470	135	700	1110	0	22	37.5	22.5	600	52.49
58	470	135	700	1110	0	22	37.5	22.5	800	25.72
59	326	184	659	1124	58	3	0	0	20	95.8
60	326	184	659	1124	58	3	0	0	650	57.9
61	326	184	659	1124	58	3	0	0	800	40
62	326	184	659	1124	58	3	0	0	950	21.3
63	391	179	689	1172	69	3.5	0	0	20	114.4
64	391	179	689	1172	69	3.5	0	0	400	84.8
65	391	179	689	1172	69	3.5	0	0	800	36.8
66	391	179	689	1172	69	3.5	0	0	950	25.4
67	442	166	689	1125	78	5.3	0	0	20	115.1
68	442	166	689	1125	78	5.3	0	0	400	85.2
69	442	166	689	1125	78	5.3	0	0	650	73.5
70	442	166	689	1125	78	5.3	0	0	950	25.5
71	440	149	702	1099	110	6.6	0	0	20	133.6
72	440	149	702	1099	110	6.6	0	0	400	98.1
73	440	149	702	1099	110	6.6	0	0	650	84.9
74	440	149	702	1099	110	6.6	0	0	800	43.1
75	437	170	783	1016	49	1.9	0	0	300	57.2
76	437	170	783	1016	49	1.9	0	0	400	58
77	437	170	783	1016	49	1.9	0	0	500	47.2
78	437	170	783	1016	49	1.9	0	0	600	36.5
79	437	170	783	1016	49	1.9	0	0	700	28.3
80	500	150	630	1260	0	10	0	0	22	49
81	500	150	630	1260	0	10	0	0	300	41
82	500	150	630	1260	0	10	0	0	400	23
83	500	150	630	1260	0	10	0	0	600	8
84	500	150	630	1260	0	10	0	0	800	3
85	450	150	630	1260	0	10	50	0	22	52
86	450	150	630	1260	0	10	50	0	105	53
87	450	150	630	1260	0	10	50	0	400	27
88	450	150	630	1260	0	10	50	0	600	11
89	450	150	630	1260	0	10	50	0	800	6
90	425	150	630	1260	0	12.5	75	0	22	57
91	425	150	630	1260	0	12.5	75	0	105	66
92	425	150	630	1260	0	12.5	75	0	300	61
93	425	150	630	1260	0	12.5	75	0	600	21
94	425	150	630	1260	0	12.5	75	0	800	12
95	400	150	630	1260	0	15	100	0	22	64
96	400	150	630	1260	0	15	100	0	105	78
97	400	150	630	1260	0	15	100	0	300	65
98	400	150	630	1260	0	15	100	0	400	37
99	400	150	630	1260	0	15	100	0	800	21
100	308	185	933	968	0	6	0	0	20	37.5
101	308	185	933	968	0	6	0	0	100	31.5
102	308	185	933	968	0	6	0	0	150	29.4
103	308	185	933	968	0	6	0	0	200	29.2
104	308	185	933	968	0	6	0	0	250	34.7
105	310	186	940	976	0	7.7	31	0	20	44.5
106	310	186	940	976	0	7.7	31	0	50	44.3
107	310	186	940	976	0	7.7	31	0	150	46.5
108	310	186	940	976	0	7.7	31	0	200	48.9
109	310	186	940	976	0	7.7	31	0	250	47.1
110	512	154	711	1106	0	18	0	0	20	80.6
111	512	154	711	1106	0	18	0	0	50	80.5
112	512	154	711	1106	0	18	0	0	100	67.8
113	512	154	711	1106	0	18	0	0	200	78.9
114	512	154	711	1106	0	18	0	0	250	83.7
115	511	153	709	1122	0	20.4	51	0	20	85.1
116	511	153	709	1122	0	20.4	51	0	50	85.2
117	511	153	709	1122	0	20.4	51	0	100	89.6
118	511	153	709	1122	0	20.4	51	0	150	94.6
119	511	153	709	1122	0	20.4	51	0	250	101.3
120	500	150	750	1068	0	0	0	0	100	75.3
121	500	150	750	1068	0	0	0	0	200	68.9
122	500	150	750	1068	0	0	0	0	400	66
123	500	150	750	1068	0	0	0	0	600	35.4
124	350	150	750	1023	150	0	0	0	23	75.2
125	350	150	750	1023	150	0	0	0	200	73.3
126	350	150	750	1023	150	0	0	0	400	60.4
127	350	150	750	1023	150	0	0	0	600	39.2
128	475	150	750	1065	0	25	0	0	23	75.7
129	475	150	750	1065	0	25	0	0	100	75.4
130	475	150	750	1065	0	25	0	0	400	68.5
131	475	150	750	1065	0	25	0	0	600	34.2
132	390	195	585	1209	0	0	0	0	23	34.1
133	390	195	585	1209	0	0	0	0	100	35.6
134	390	195	585	1209	0	0	0	0	200	31.6
135	390	195	585	1209	0	0	0	0	600	16.8
136	390	195	585	1209	0	0	0	0	400	26.6
137	572	286	1345	0	0	0	0	0	600	43.4
138	786	236	1286	0	0	25.9	78.6	0	800	41.3
139	572	286	1345	0	0	0	0	0	23	58.3
140	572	286	1345	0	0	0	0	0	200	55
141	572	286	1345	0	0	0	0	0	400	52.2
142	572	286	1345	0	0	0	0	0	800	31.5
143	572	286	1345	0	0	0	0	0	1000	6.5
144	786	236	1286	0	0	25.9	78.6	0	23	71
145	786	236	1286	0	0	25.9	78.6	0	200	58
146	786	236	1286	0	0	25.9	78.6	0	400	65.4
147	786	236	1286	0	0	25.9	78.6	0	600	62.9
148	786	236	1286	0	0	25.9	78.6	0	1000	21
149	430	172	687	1030	0	1.6	0	0	20	61.8
150	430	172	687	1030	0	1.6	0	0	100	53.3
151	430	172	687	1030	0	1.6	0	0	200	55.5
152	430	172	687	1030	0	1.6	0	0	300	46.5
153	430	172	687	1030	0	1.6	0	0	600	20.6
154	441	164	653	1115	0	2.9	28	0	100	62.8
155	441	164	653	1115	0	2.9	28	0	200	64.7
156	441	164	653	1115	0	2.9	28	0	300	56.5
157	441	164	653	1115	0	2.9	28	0	600	21.8
158	495	149	615	1168	0	1.9	0	0	20	67.4
159	495	149	615	1168	0	1.9	0	0	200	59.7
160	495	149	615	1168	0	1.9	0	0	300	49
161	495	149	615	1168	0	1.9	0	0	600	21
162	465	149	615	1168	0	3.1	30	0	20	80.3
163	465	149	615	1168	0	3.1	30	0	100	68
164	465	149	615	1168	0	3.1	30	0	300	56.5
165	465	149	615	1168	0	3.1	30	0	600	23.4
166	450	149	615	1168	0	3.7	45	0	20	84.2
167	450	149	615	1168	0	3.7	45	0	100	70.8
168	450	149	615	1168	0	3.7	45	0	200	71.7
169	250	123	417	1681	0	0	0	0	100	25.74
170	350	172	373	1507	0	0	0	0	200	40.35
171	500	385	0	820	0	6	0	0	400	34.5
172	450	346.5	0	805	0	6	50	0	800	21
173	400	308	0	790	0	6	100	0	200	44
174	350	269.5	0	775	0	6	150	0	400	27
175	400	308	0	1038	0	4.8	0	0	800	7.5
176	360	277.2	0	1028	0	4.8	40	0	200	32
177	320	246.4	0	1015	0	4.8	80	0	400	30
178	280	215.6	0	1005	0	4.8	120	0	800	8.5
179	500	135	700	1110	0	14	30	0	400	69.87
180	500	135	700	1110	0	15	22.5	7.5	600	45.23
181	500	135	700	1110	0	16	15	15	600	48.79
182	500	135	700	1110	0	18	7.5	22.5	800	27.38
183	470	135	700	1110	0	16	60	0	400	69.86
184	470	135	700	1110	0	18	52.5	7.5	600	47.07
185	470	135	700	1110	0	20	45	15	800	22.32
186	470	135	700	1110	0	22	37.5	22.5	20	91.24
187	326	184	659	1124	58	3	0	0	400	69.2
188	391	179	689	1172	69	3.5	0	0	650	66.9
189	442	166	689	1125	78	5.3	0	0	800	37.9
190	440	149	702	1099	110	6.6	0	0	950	29.4
191	437	170	783	1016	49	1.9	0	0	20	71.2
192	500	150	630	1260	0	10	0	0	105	51
193	450	150	630	1260	0	10	50	0	300	49
194	425	150	630	1260	0	12.5	75	0	400	32
195	400	150	630	1260	0	15	100	0	600	28
196	308	185	933	968	0	6	0	0	50	37.2
197	310	186	940	976	0	7.7	31	0	100	44.1
198	512	154	711	1106	0	18	0	0	150	72.8
199	511	153	709	1122	0	20.4	51	0	200	95.3
200	500	150	750	1068	0	0	0	0	23	75.5
201	350	150	750	1023	150	0	0	0	100	73.7
202	475	150	750	1065	0	25	0	0	200	73.4
203	441	164	653	1115	0	2.9	28	0	20	73.9
204	495	149	615	1168	0	1.9	0	0	100	57.6
205	465	149	615	1168	0	3.1	30	0	200	69
206	450	149	615	1168	0	3.7	45	0	300	57.9
207	450	149	615	1168	0	3.7	45	0	600	22.6
